# Cognitive and neural principles of a memory bias on preferential choices

**DOI:** 10.1016/j.crneur.2022.100029

**Published:** 2022-02-08

**Authors:** Peter M. Kraemer, Regina A. Weilbächer, Tehilla Mechera-Ostrovsky, Sebastian Gluth

**Affiliations:** a Department of Psychology, University of Basel, Switzerland; b Department of Psychology, University of New South Wales, Australia; c Department of Psychology, University of Hamburg, Germany

**Keywords:** Value-based decision making, Episodic memory, Ventromedial prefrontal cortex, Hippocampus, fMRI, Cognitive modeling

## Abstract

Value-based decisions depend on different forms of memory. However, the respective roles of memory and valuation processes that give rise to these decisions are often vaguely described and have rarely been investigated jointly. In this review article, we address the problem of memory-based decision making from a neuroeconomic perspective. We first describe the neural and cognitive processes involved in decisions requiring memory processes, with a focus on episodic memory. Based on the results of a systematic research program, we then spotlight the phenomenon of the memory bias, a general preference for choice options that can be retrieved from episodic memory more successfully. Our findings indicate that failed memory recall biases neural valuation processes as indicated by altered effective connectivity between the hippocampus and ventromedial prefrontal cortex. This bias can be attributed to meta-cognitive beliefs about the relationship between subjective value and memory as well as to uncertainty aversion. After summarizing the findings, we outline potential future research endeavors to integrate the two research traditions of memory and decision making.

## Highlights

• Episodic memory influences choices as people prefer better-remembered options.
• Interplay between hippocampus and ventromedial prefrontal cortex promotes this memory bias.
• Choice uncertainty and meta-cognitive beliefs about memory mediate the memory bias.
• Strong influence of attention on valuation in decisions that are based on memories.

## Graphical abstract

**Figure:**
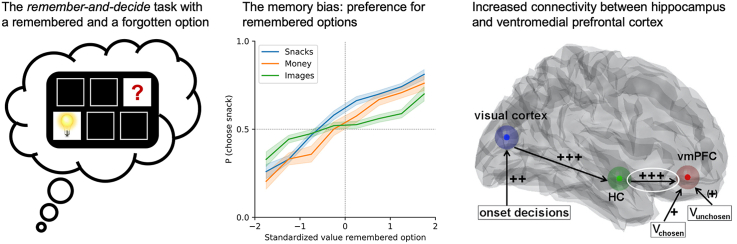

## 1 Introduction

Most of our daily decisions rely on past experiences. Be it what food we prefer, whether to invest in stocks or what pictures we like on social media, we never face these decisions with a clean sheet. Instead, we are influenced by our past learning, semantic knowledge, or autobiographical background. In other words: Decision making strongly depends on information that we obtain from memory. But how do we make decisions when memory recall is impaired? How do we decide when we do not recall how a specific food tastes, how well a stock has performed in the past, or what content a picture shows? How do our decision processes adapt to such situations when memory fails?

In this review article, we first give a neuroeconomic perspective on value-based decision processes and how they relate to memory on a cognitive and neural level. Second, we review the literature on how people make decisions when significant information about the potential choice options cannot be made available by memory processes. Third, we spotlight the *memory bias*, a human tendency to prefer even relatively unattractive choice options over uncertain options that cannot be adequately recalled. Fourth, we summarize the current state of research regarding the memory bias and connect it to recent literature on the interplay of memory and decision making. Finally, we describe future avenues and challenges for studying the cognitive neuroscience of memory-based decision making.

## 2 Decision making and memory

### 2.1 A neuroeconomic perspective on decision making

How do we decide between different alternatives? Over centuries, mathematicians, economists, and later on also psychologists and neuroscientists have been concerned with this question. The dominant view nowadays comes from expected utility theory, proposing that decision-makers decide between different options as if they seek to maximize the utility they derive from doing so (von Neumann and Morgenstern, 1953). An alternative term for utility is *subjective value*, defined as a latent variable that describes the personal preference for a choice option (Menger, 1871). We will use the term subjective value for the remainder of this review. For decades, researchers treated subjective value as a black box that cannot be observed but only inferred from choice behavior (Samuelson, 1937). However, the relatively young discipline of neuroeconomics draws on theory and methodology from economics, cognitive psychology, and neuroscience to gain a deeper understanding of how subjective value is computed and used for decision making (Camerer et al., 2005; Glimcher and Fehr, 2013).

A popular model to study value-based choice is the two-stage model (e.g., Platt and Plassmann, 2013), according to which decision making occurs essentially in the two stages of *valuation* and *action selection*. During valuation, decision-makers attribute subjective value to the choice options they consider, thereby forming a neural representation of value. Converging evidence from neurophysiological and lesion studies, some of which date back to the 1980s (for overviews, see Montague and Berns, 2002; Rolls, 2000), as well as from ensuing brain imaging studies (Bartra et al., 2013; Clithero and Rangel, 2014) suggest frontal brain regions, particularly the ventromedial prefrontal cortex (vmPFC), to show an activity profile that matches the idea of a representation of subjective value. vmPFC seems to construct and integrate value signals (Bault et al., 2019; Chib et al., 2009; Vaidya and Fellows, 2020) and contribute to the comparison between value-based choice options (Boorman et al., 2009; Rushworth et al., 2011).

During action selection, decision-makers consider alternative action plans (e.g., approaching one or another choice option) and select among them to achieve a current goal (e.g., maximize subjective value). Several cortical regions such as the posterior parietal cortex, the frontal eye fields, or the supplementary motor area have been associated with the representation and selection of action plans (see, Cisek and Kalaska, 2010; Gold and Shadlen, 2007, for reviews). As association areas, these regions receive afferent inputs from various brain areas involved in perception, cognition, and valuation and have efferent connections with motor areas that initiate the potential actions (Shadlen and Shohamy, 2016). Neurons in the association areas are effector-specific and tuned to particular actions (Cisek, 2007). Action selection can be understood as the competitive activation of these neurons over time. When the activation level of one action plan outweighs that of all others by some margin, the respective behavior is triggered, much like in an accumulation-to-bound process (Shadlen and Kiani, 2013). While in perceptual decisions, action selection draws on sensory inputs, in value-based decisions, regions that code for subjective value affect action selection (Busemeyer et al., 2019; Hunt and Hayden, 2017; Shadlen and Shohamy, 2016).

The two-stage model states that valuation and action selection are two critical processes for value-based decision making (Platt and Plassmann, 2013; Rangel et al., 2008). Choice options need to be evaluated to represent subjective value. Further, the subjective value of different options needs to be compared to select an appropriate action. It is debated whether the process of comparing subjective values of different options is executed in regions such as vmPFC that code for the value of choice options (Padoa-Schioppa, 2011; Rushworth et al., 2012) or in regions that code for actions (Cisek and Kalaska, 2010; Gold and Shadlen, 2007). While the solution of this debate is not directly relevant to our work, it highlights the importance of a detailed understanding of how subjective value is assessed and computed in the neuro-cognitive system. We argue that valuation crucially depends on memory processes, as discussed below.

### 2.2 Why memory is important for decision making

There is consensus that value signals in the brain are constructed with the support of memory (Hunt and Hayden, 2017; Shadlen and Shohamy, 2016; Shohamy and Daw, 2015). Although the interplay of memory and value-based decision processes was recognized decades ago in the literature (Lynch and Srull, 1982; Nedungadi, 1990), the two cognitive functions have been studied largely in separation. This, however, has changed recently, as neuroeconomists have become increasingly interested in understanding the role of memory-related brain regions such as the hippocampus in value-based choice tasks (Shohamy and Daw, 2015). At the same time, psychological research developed more integrative models of judgment and decision making (Johnson et al., 2007; Stewart et al., 2006; Suri et al., 2020; Zhao et al., 2021). Also, behavioral economics started to integrate memory processes into their theories in order to explain relevant contextual biases on consumer and investment behavior (Bordalo et al., 2020; Gödker et al., 2019).

Importantly, memory is not a homogeneous cognitive function but an umbrella term that comprises several, loosely connected mechanisms and systems. To elaborate on how value is constructed from memory, it is necessary to understand what form of memory is under consideration. Classically, cognitive psychology distinguishes between two major long-term memory systems: declarative and non-declarative memory (Cohen and Squire, 1980). Non-declarative memory describes implicit memory processes which cannot be verbalized, such as procedural memory (skills and habits), priming, classical conditioning, and non-associative learning. On the other hand, the declarative memory system circumscribes semantic memory for facts about the world and episodic memory for past experienced events (Milner et al., 1998). Retrieval of episodic memories is characterized by a conscious recollection of the events alongside the context in which they took place. Although episodic memory covers autobiographical episodes, it is not to be confused with autobiographical memory, which focuses on autobiographical contents without a conscious recollection of context (Wheeler et al., 1997) and has a partly different neural basis (Gilboa, 2004).

Traditionally, neuroeconomists focused on the impact of non-declarative memory processes and, in particular, reinforcement learning on decision making (see Dayan and Niv, 2008; Dolan and Dayan, 2013, for a review). This framework is concerned with how decision-makers learn action-dependent habits, which can affect the probabilities of specific responses without an explicit representation of value (Mishkin et al., 1984). In recent years, however, the field has become more and more interested in how decision-makers weigh in mnemonic information from episodic memory (Biderman et al., 2020; Shadlen and Shohamy, 2016; Weilbächer & Gluth, 2017). Accordingly, people retrieve specific episodes from past rewarding or punishing events and integrate these into their valuation processes when making new decisions. Notably, episodic memory has also been implicated in reinforcement learning (Bornstein et al., 2017; Gershman and Daw, 2017) and decisions from experience (Hertwig et al., 2004), both being prominent research directions in neuroeconomics.

On the neural level, a long tradition of lesion and neuroimaging studies identified the hippocampus and the surrounding medial temporal lobe as key players in episodic memory (see Milner et al., 1998; Squire and Wixted, 2011, for reviews). In value-based decisions from memory, it seems that the hippocampus engages with the vmPFC to affect valuation processes, as shown by electrophysiological and brain imaging studies (see Palombo et al., 2015; Weilbächer & Gluth, 2017, for reviews). Thus, during a decision between several options, episodic memories that are associated with those options are being recalled and affect their valuation (Bakkour et al., 2019; Murty et al., 2016), which in turn drives the action selection process (Biderman et al., 2020; Shadlen and Shohamy, 2016).

On the other hand, the hippocampus and the surrounding medial temporal lobe are not the only brain regions involved in episodic memory, and the vmPFC has not only been associated with decision-making but also with memory-related processes. For example, patients with vmPFC lesions construct past and future events only with low episodic detail (Bertossi et al., 2016), and lesions to the mPFC in rats lead to impairments of recalling place-reward associations (Seamans et al., 1995). Furthermore, brain imaging studies have identified the parietal lobe as another critical brain region that is regularly involved in episodic memory tasks (Wagner et al., 2005). An interesting suggestion concerning this area's role in episodic memory is that it may implement an accumulation-to-bound process for memory retrieval, given that activity in the lateral posterior parietal cortex is tightly coupled with accuracy and response times in recognition tasks (Sestieri et al., 2014; Wagner et al., 2005). In other words, neurons in the parietal lobe may accumulate evidence for identifying an object as recognized or novel. Given all of these findings, the neural circuitry that mediates value-based decisions from memory is arguably much more complex than the mere assumption of a hippocampus-to-vmPFC axis, in which the former region would strictly represent mnemonic processes, and the latter would be solely associated with choice-related mechanisms.

## 3 When memory fails to inform decisions

The relationship of memory and decision making can be better understood by adopting the view of humans as bounded-rational decision makers (Kahneman and Tversky, 1979; Simon, 1956) who have to adapt their choice strategies to meet the challenges of limited knowledge and cognitive capacities. From this perspective, it is reasonable to assume that some violations of classic economic theory originate from limits associated with the cognitive process of memory retrieval (Weber and Johnson, 2006).

For example, the impact of memory retrieval on decisions from experience has been examined extensively (Hertwig et al., 2004). In these tasks, people learn about the potential outcomes of choice options by receiving feedback for chosen options. Many of those decisions are affected by the accessibility of information in memory. For instance, the serial position of information affects memory retrieval and choice. First presented information (i.e., primacy effect) and most recently learned information (i.e., recency effect) have been shown to have a greater likelihood to be remembered and to influence choice (Hertwig et al., 2004; Shteingart et al., 2013). Madan et al. (2014) showed that associative priming (Ludvig et al., 2015), which describes the increased likelihood of retrieving items associated with extreme outcomes (positive or negative), exerts an effect on risky choices. Similarly, St-Amand et al., 2018 showed that, in decision from experience, people become more risk averse when episodic memory is attenuated, that is, they choose the option that always offered the same amount of reward more frequently.

A further pervasive role of memory was shown in consumer choices. Nedungadi (1990) showed that priming affects information retrieval and biases brand choices. Food choices are affected by the neurological decline of memory-related brain regions due to age (Levin et al., 2019) and brain damage (Bakkour et al., 2019; Enkavi et al., 2017). Taken together, these studies emphasize the importance of accessibility of information in memory-based decision making.

### 3.1 The memory bias

The role of accessibility of memory content becomes evident when people fail to access memory during choice formation. Empirical work has shown that decision makers adapt to such situations by shifting their preferences towards the options they can remember better. This is true even if the (better) remembered options are comparatively unattractive (Gluth et al., 2015). Thus, people are willing to accept relatively bad options as long as they have good memory for them. To give an everyday example, people may choose to eat at an arguably unattractive fast-food restaurant instead of an alternative one, because they might recall less details from their previous visits of the latter compared to the former. Although chances are that the alternative restaurant is preferable, people tend to prefer the unattractive option which is associated with more detailed memory recall. Gluth et al. (2015) termed this effect the *memory bias*.

**Fig. 1:**
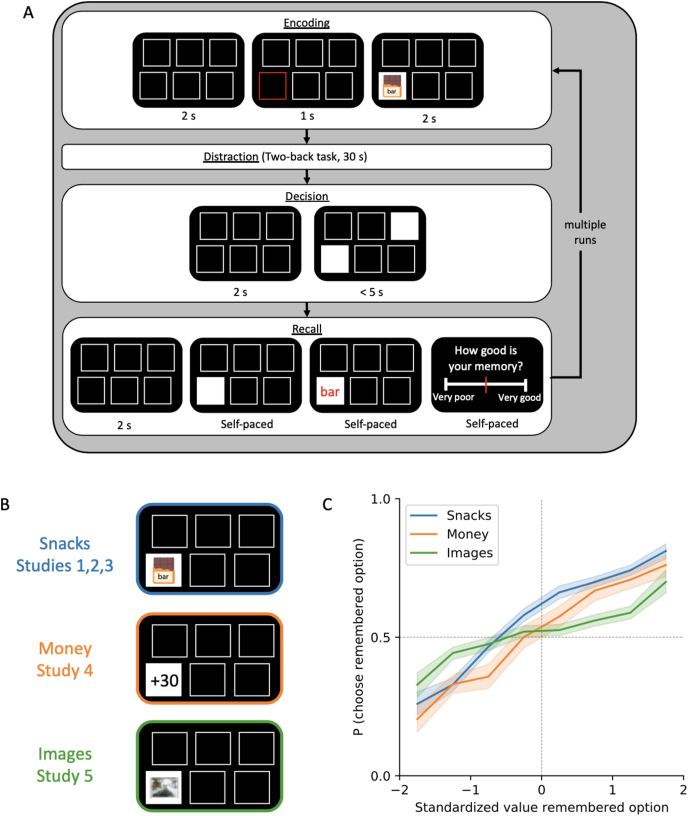
Remember-and-decide task. **A)** Task structure with periods Encoding, Distraction, Decision and Recall. The core element of the task is the requirement to recall the options when making decisions. **B)** Different stimulus categories used in the experiments. **C)** Choice data, showing the memory bias. The curves show the probability of choosing the remembered option (as a function of its standardized subjective value) when the other option cannot be recalled. The memory bias is evident by the fact that the psychometric curves cross the indifference line (P = 0.5) at a standardized value below 0, reflecting an elevated preference for the remembered option. It has been replicated in multiple experiments and across different stimulus categories.

The phenomenon was established using the *remember-and-decide* task (Fig. 1A). This task comprises several rounds, each consisting of four phases: encoding, distraction, decision and cued recall. During the *encoding phase*, participants learn the association between a number of items (e.g., food snacks, images, monetary rewards) and their location on the screen. Next, a *distraction phase* prevents participants from maintaining the encoded item-location associations in their working memory. In the *decision phase*, participants face a two-alternative forced choice task. In each decision trial, two locations from the encoding phase are highlighted and participants decide which item they prefer. Importantly, the items are “hidden” so that the items’ identities need to be recalled from memory. After performing a number of these decision trials, participants enter the recall phase in which their ability to retrieve the items behind each location is tested. For each of these items, participants then rated the memory strength, that is, they reported their subjective estimation of vividness of the snack in memory.

The core feature of the remember-and-decide task is that, in the decision period, participants need to retrieve item-specific information from memory to make informed preferential choices. Obviously, choices depend heavily on whether the item can be retrieved or not. When both items are recalled, choice probability is a probabilistic function of the value difference between the two options, just as in regular value-based decisions (McFadden, 2001). When none of the items is recalled, participants are forced to guess. Most importantly, when only one option, but not the other, can be recalled, participants must solely rely on the value of the remembered option to make an informed decision. In this case, we observed a biased choice behavior as participants did not only choose remembered options of average and above-average but also of below-average subjective value. Only remembered options of extremely low subjective value were rejected. This *memory bias* can be visualized as a shift in the choice curve that links the standardized subjective value of the remembered option to its probability of being chosen (Fig. 1C).

Across five studies, we investigated the cognitive and neural mechanisms that mediate the memory bias. After identifying the hippocampus and vmPFC as key brain regions (Gluth et al., 2015), we assessed to what extent the memory bias is driven by subjective beliefs in memory strength (Mechera-Ostrovsky and Gluth, 2018), visual attention (Weilbächer et al., 2021), and whether the memory bias resembles decisions under uncertainty (Weilbächer et al., 2020). Finally, we tested whether the bias is better accounted for by a single evaluation process or by dual processes involving heuristics and utility maximization (Kraemer et al., 2020). In the next section, we revisit this work and draw connections from it to related recent literature on memory-based decision making.

### 3.2 The neural basis of the memory bias (Study 1)

Gluth et al. (2015) discovered the memory bias in a fMRI study with three groups of participants (two memory groups, one control group) performing the remember-and-decide task with food snacks as choice options. While the two memory groups exhibited the memory bias, the control group –which did not have to recall but saw the snacks during the decision phase– did not show a tendency to choose the options they could recall in the ensuing recall period.

**Fig. 2:**
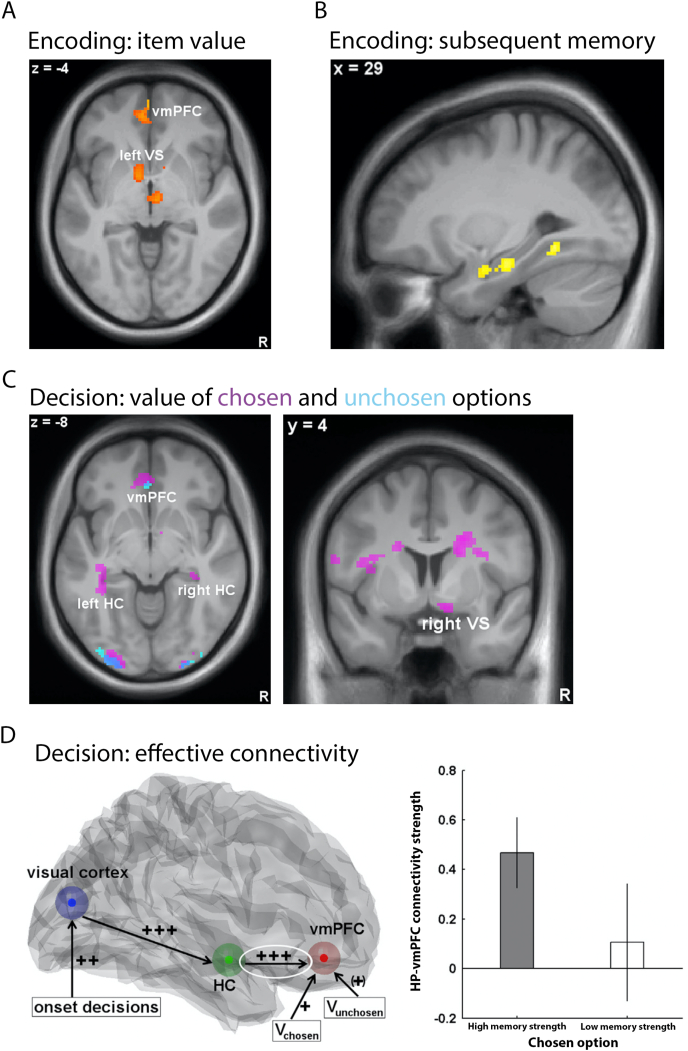
Neural mechanisms of memory-based decisions (Gluth et al., 2015). **A** and **B)** During encoding of future choice options, vmPFC (together with ventral striatum; VS) represents the subjective value of the options, and hippocampus predicts which item will be remembered later on. **C)** During memory-based decisions, both vmPFC and hippocampus (HC) represent the value of chosen options, but only vmPFC encodes unchosen option values as well. **D)** A significantly positive coupling from HC to vmPFC was only observed in trials, in which the better remembered option was chosen, suggesting that this connectivity mediates the memory bias.

As participants encoded the snack-location associations, ventral striatum and vmPFC activity was correlated with the subjective value of the snacks (Fig. 2A). Both areas are projection areas of the dopaminergic reward system and encode subjective value (Bartraet al., 2013). While the hippocampus was not sensitive to subjective value during encoding, its activity predicted whether a snack was recalled during decision and recall phases, in line with subsequent memory effects (Kim, 2011) (Fig. 2B). During the decision phase, the snack values of chosen options, modulated by their memory strength, were present in vmPFC and anterior hippocampus, but the value of unchosen options were only correlated with vmPFC activity (Fig. 2C). These results suggest that the hippocampus was partially co-activated with the valuation system during memory-based decisions, in line with previous evidence for representations of subjective value in the hippocampus (Lebreton et al., 2009; H. Lee et al., 2012). When testing for a neural correlate of the memory bias, an increased hippocampus-vmPFC connectivity was found when participants chose the better remembered option (Fig. 2D). Thus, the hippocampus-vmPFC axis appears to be more involved when memory strength has an effect on choice. This could implicate that the hippocampus affects valuation processes in vmPFC in a way which biases action selection in favor of remembered options (Shadlen and Shohamy, 2016). This is in line with converging literature on the role of the hippocampus in deliberation (Bakkour et al., 2019; Biderman et al., 2020; Bornstein and Norman, 2017).

These findings suggest that valuation processes in vmPFC depend on hippocampus when episodic memories affect deliberation. Interestingly, a recent study by Zhang et al. (2021) found functional coupling of vmPFC and antero-lateral prefrontal cortex when semantic memory is used, presumably for choice set generation and semantic retrieval. It is an open question whether such decisions would also be affected by a memory bias. For instance a memory bias could play out as a brand effect, where people choose brands that they know, even if the product quality may be arguably low. This could be due to a higher association of a brand with its related features as compared to a retrieved alternative.

### 3.3 The role of meta-cognitive beliefs about memory (Study 2)

Having gained insight on its neural mechanisms, further questions arose pertaining to the exact psychological mechanisms that drive the memory bias. First, we examined whether meta-cognitive beliefs about memory strength play a role in the memory bias. According to this rationale, when participants can only remember one option they may discount the other (not-remembered) option for the very reason that they do not remember it. Psychologically speaking, one could think of this mechanism as an “if it's not remembered, it must be bad” heuristic. Hence, we derived the hypothesis that a stronger belief in the value-dependency of memory strength should correlate positively with the memory bias.

To test this hypothesis, we conducted a preregistered experiment that included not only the remember-and-decide task but also a second task that assessed participants’ beliefs about their performance in the remember-and-decide task (Mechera-Ostrovsky and Gluth, 2018). In this *estimate-your-memory task*, participants indicated for every snack how often they were able to recall it. In addition to replicating the memory bias, we found that snacks of high subjective value were indeed estimated to be recalled more often. Most importantly and as predicted, there was a significantly positive correlation between this value-dependency of beliefs and the memory bias on choice. This suggests that meta-cognitive beliefs about the relationship between subjective value and memory contribute to our preference for better-remembered alternatives. Future work should investigate the neural mechanisms that underlie this contribution. In particular, it would be interesting to know whether the connectivity between hippocampus and vmPFC in memory-based decisions is modulated by regions such as the rostrolateral prefrontal cortex, which has been linked to meta-cognitive assessments in perceptual and value-based choice (De Martino et al., 2013; Fleming et al., 2012).

Beyond this effect of subjective value on (subjective) beliefs, a non-linear relationship between value and (objective) memory performance was found. That is, participants showed a higher recall probability for items with very high values but also (to a lesser extent) for items with very low values. Intriguingly, it has been shown that both confidence judgments in value-based decisions (Lebreton et al., 2015) and meta-cognitive judgments of mnemonic performance (Hebscher et al., 2016) exhibit similar U-shaped relationships with subjective value and recognition accuracy, respectively. Taken together, these results seem to indicate that the memory bias is tightly linked to the notion of confidence on both the cognitive and the neural level. More specifically, when choosing between a remembered and a forgotten option, people may take into account whether they have sufficient memory strength for choosing an option. Because this memory strength (or confidence) appears to be lowest for options that are slightly below average in terms of subjective value, as seen in this study, the decision is most difficult at this point, which is in line with people being indifferent between the two options.

### 3.4 The role of attention (Study 3)

Recent research suggests that overt visual attention affects value comparisons in value-based choice (Krajbich, 2019). Although it is currently debated whether value or choice is modulated (Mormann and Russo, 2021), there is a robust empirical effect that people tend to choose options they have looked at longer (Fiedler & Glöckner, 2012; Gluth et al., 2020; Krajbich et al., 2010; Pärnamets et al., 2015; Stewart et al., 2016; Westbrook et al., 2020). On the other hand, looking behavior is also linked to memory retrieval processes (see, Lai et al., 2013, for a review). In experimental situations, it is established that people look at the locations of previously presented options while they retrieve information from memory (Renkewitz and Jahn, 2012; Scholz et al., 2015). Since overt attention is related to retrieval and to decision processes, we asked whether overt attention contributes to the memory bias.

To investigate this, Weilbächer et al. (2021) used eye tracking to record gaze patterns in the remember-and-decide task. Once more, we replicated the behavioral effect of the memory bias. In line with the studies mentioned above, we also found that chosen options received significantly more attention than unchosen options. However, remembered options were not looked at longer or more often than not-remembered options. Thus, both an attention bias and a memory bias were present, but they did not influence each other. More generally, the study allowed us to compare decisions with visually presented options (value trials) against decisions that required options to be recalled from memory (memory trials). We found that the influence of attention on choice was stronger in memory trials compared to value trials. We speculate that in the case of memory-based decisions, attention is likely to be relevant for the retrieval process itself, potentially facilitating information retrieval (Richardson and Spivey, 2000; Scholz et al., 2011). As a consequence, people may exhibit an even stronger preference for attended options in these kind of decisions. Additionally, we found that decisions involving episodic memory were less consistent with subjective value ratings. This higher stochasticity likely depends on the fact that information needs to be retrieved from episodic memory. Some theories of decision making suggest that subjective values are sampled or retrieved from internal value representations (Polanía et al., 2019; Stewart et al., 2006). It seems plausible that the greater stochasticity is due to uncertain success of the memory retrieval or sampling processes. If this is true, then choice consistency should be tightly coupled to memory strength. Future studies may further look into this relationship, for instance, by varying memory strength in a parametric way.

### 3.5 The role of uncertainty (Study 4 and 5)

Arguably the most obvious cognitive mechanism underlying the memory bias is uncertainty. Specifically, we reasoned that people dislike choosing an option they have no or only little memory of, because they are uncertain about that option's subjective value. Given this rationale, memory-based decisions share conceptual similarities with decisions under uncertainty. The latter are typically studied with lottery gambles and can entail both risk (when the probability of an outcome is known) and ambiguity (when the probability is unknown). As discussed in the introduction, economists and behavioral scientists have studied decision making under risk and ambiguity extensively (Oppenheimer and Kelso, 2015) and have identified many puzzling behavioral effects. One of these effects is the *reflection effect*, the finding that people are less willing to accept uncertainty when facing potential gains compared to potential losses (Kahn and Sarin, 1988; Kahneman and Tversky, 1979; Tversky and Kahneman, 1981; Viscusi and Magat, 1992). Connecting memory- and uncertainty-based decisions, we thus predicted that the tendency to prefer better-remembered options (i.e., the memory bias) should be less pronounced in the loss as compared to the gain domain. In Weilbächer et al. (2020), we ran two preregistered experiments in which participants performed the remember-and-decide task in the gain as well as the loss domain. The two experiments differed in terms of stimulus material (positive and negative monetary values; appetitive and aversive pictures). Confirming our prediction and thus the link between decisions from memory and decisions under uncertainty, we found that the memory bias was less pronounced in the loss domain compared to the gain domain. In other words, when facing potential gains, people stick to better-remembered options and avoid the risk of choosing what they do not recall well, but when it comes to potential losses, people take the risk and are more likely to choose the unknown. A potential explanation may be linked to the above-mentioned finding that the memory bias depends on hippocampal-vmPFC connectivity (Fig. 2D). Assuming that the hippocampus biases valuation in vmPFC, this bias may be less relevant in the loss domain since the vmPFC seems to be less sensitive to negative values (Bartra et al., 2013). Another possibility is that regions known to mediate framing effects and uncertainty in value-based decisions such as the amygdala and the orbitofrontal cortex (De Martino et al., 2006; Hsu et al., 2005) modulate the connection between hippocampus and vmPFC to promote the memory bias.

### 3.6 Decision processes underlying the memory bias

In the previous sections, we demonstrated that the memory bias is a robust empirical finding, which generalizes over several stimulus domains and can at least partially be attributed to subjective beliefs about memory strength as well as to uncertainty-related preferences. While these factors outline potential reasons for the occurrence of the memory bias, the computational mechanisms which give rise to this phenomenon remain unknown. For instance, it is conceivable that people exhibit the memory bias because they may rely on a heuristic choice process that favors choosing remembered options (note that the underlying cognitive process, such as a memory-dependent heuristic, is not to be confused with the empirical phenomenon of the memory bias itself). Analogous to the recognition heuristic, according to which people make judgements simply based on whether they recognize an option or not (Goldstein and Gigerenzer, 2002), participants in our studies may sometimes have decided simply based on whether they have remembered an option or not. In the section on subjective beliefs (see above), we argued that a heuristic process alone cannot explain the memory bias, because people rejected remembered options with very low values. However, heuristic processes have been suggested to be used adaptively from a wider repertoire of decision processes (Marewski and Link, 2014; Pachur, 2011). From a perspective of dual-process theories, people may thus alternate between fast and erroneous heuristic decisions and slower but more accurate decision processes that entail higher cognitive effort (Alós-Ferrer, 2018; Evans, 2008; Kahneman, 2011). In terms of memory-based choices, people may resort sometimes to a recognition-like heuristic and at other times to a utility-maximizing but cognitively costly process. Such a dual-process account would result in a general tendency to choose remembered options while giving the flexibility to reject very low-valued options.

In contrast to dual-process theories, Gluth et al. (2015) suggested that a single choice mechanism is sufficient to explain the memory bias. According to this single-process account, people accumulate preference by comparing the subjective value of the remembered option with a reference value, which acts as a replacement of the unavailable value of the not-remembered option. This reference value is biased so that participants tend to prefer remembered options. In Kraemer et al. (2020), we tested whether this single-process model or a dual-process account that alternates between a heuristic and a utility mechanism explains the memory bias best. The challenge here was that both, single- and dual-process accounts predicted a choice pattern which resembled the behavior of the memory bias. Yet, we were able to show that the two accounts differ with respect to their response time (RT) predictions. Resorting to the data by Mechera-Ostrovsky and Gluth (2018), we adopted hierarchical Bayesian modeling techniques (M. D. Lee and Wagenmakers, 2013) to compare the two accounts. Qualitative as well as quantitative model comparisons provided strong support for the single-process account, as it was more in line with the RT predictions. Thus, we conclude that the memory-bias depends on a single biased evaluation process rather than an alternation between heuristic and utility-maximizing strategies.

**Fig. 3:**
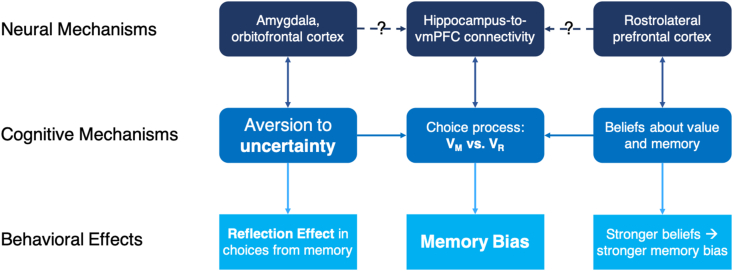
The knowns and unknowns of the neural and cognitive mechanisms underlying the memory bias. Cognitively, the memory bias arises from a (biased) comparison process between the value of the memorized option (V_M_) and and a reference value (V_R_) that replaces the value of the not-remembered option. We have shown that the phenomenon is related to an increased hippocampus-to-vmPFC connectivity, and that uncertainty and beliefs about memory contribute to it. What is currently unknown, however, are the neural mechanism underling these influences of uncertainty and beliefs.

As a summary of the current state of knowledge and the open questions surrounding the memory bias, Fig. 3 provides a schematic overview of what we currently know and still do not know about the neural and cognitive mechanisms underlying this influence of memory on value-based decisions.

## 4 Perspectives

In the previous sections, we have argued that valuation processes often depend on episodic memory which is consequential for value-based decision making. This relation becomes particularly critical when memory processes fail. In such cases, the neural and cognitive systems adapt to allow us to make informed decisions that are biased towards better-memorized options due to meta-cognitive beliefs and uncertainty aversion. In this concluding chapter, we outline future important research endeavors of memory-based decision making more generally.

### 4.1 Neural basis of the influence of beliefs and uncertainty

As discussed above and depicted in Fig. 3, the neural basis of the memory bias’ modulation by meta-cognitive beliefs about value, memory, and uncertainty aversion remains to be discovered. In our view, a plausible assumption is that regions that are known to be involved in meta-cognitive assessments (e.g., rostrolateral prefrontal cortex, rlPFC; De Martino et al., 2013; Fleming et al., 2012) on the one hand and decisions under uncertainty (e.g., amygdala and orbitofrontal cortex, OFC; De Martino et al., 2006; Hsu et al., 2005) on the other hand modulate memory bias by acting on the choice-related processes in the vmPFC directly or by affecting the connectivity between hippocampus and vmPFC that we have linked to the memory bias (Gluth et al., 2015).

Concerning meta-cognitive beliefs, the rlPFC has been linked to the ability to make meta-cognitive judgments about one's own performance (Fleming et al., 2010). Further, De Martino et al. (2013) have reported an increased functional connectivity between this region and the vmPFC when decisions were made with high confidence. Thus, believing in a strong relationship between subjective value and memory strength could enhance a decision-maker's confidence when choosing well-remembered options. This may be reflected in increased activity in rlPFC but also in a stronger influence of this region's activity on the choice dynamics of vmPFC.

Regarding uncertainty, activity in the amygdala appears to be higher when decisions are in alignment with the above-discussed reflection effect (i.e., choosing safe options in the gain domain but risky options in the loss domain). At the same time, activity in OFC scales with the ability to overcome this tendency (De Martino et al., 2006). Thus, a tentative hypothesis could be that these regions exert different influences on the hippocampus-vmPFC connectivity, which mediates the memory bias. The amygdala may strengthen the hippocampus-vmPFC connectivity for decisions in the gain domain but weaken it for decisions in the loss domain. On the other hand, the OFC may weaken the relationship between the hippocampus-vmPFC connectivity and the behavioral phenomenon of the memory bias in general, leading to more rational decisions (in a strictly economic understanding of rationality). These hypotheses could be investigated with neuroimaging studies that make use of Dynamic Causal Modeling (Friston et al., 2003), which allows testing whether a third region's activity modulates the connectivity between two other regions.

Additionally, it has been shown that reward-related brain regions such as the ventral striatum are more activated in the case of successful and highly confident memory retrieval (Clos et al., 2015; Schwarze et al., 2013) and when identifying previously seen items as “old” in recognition tasks compared to identifying novel items as “new” (Han et al., 2010). This has been interpreted as reflecting the pleasure of being able to retrieve information accurately. Hence, it is tempting to speculate that the joy of retrieving an option is mingled with the estimation of that option's subjective value, which should promote the memory bias. Similarly, higher confidence and associated joy in remembering snacks may activate the brain's reward system, and these reward signals could be mingled with the option's subjective value representation. As a result, the brain may overestimate the subjective value of well-remembered options, resulting in the memory bias phenomenon.

### 4.2 Sequential sampling modeling of choices from memory

Memory-based decision-making is a challenging topic to study because it involves understanding several cognitive processes (i.e., memory and decision formation). Moreover, cognitive processes are latent variables that cannot be observed directly but must be inferred from behavior and brain function. To study these processes, mathematical and computational models of cognition are useful tools (Farrell and Lewandowsky, 2018). In neuroeconomics, the sequential sampling framework appears to become the dominant paradigm to model value-based decision making and economic choice (Clithero, 2018; Fehr and Rangel, 2011). As briefly mentioned above, the core idea of this framework is that decisions arise from a noisy process of sampling or accumulating pieces of evidence in a sequential manner, which is terminated as soon as the desired level of evidence or confidence about the most appropriate choice option has been collected. In several of our own studies (Gluth et al., 2015; Kraemer et al., 2020; Weilbächer et al., 2021), we adopted the sequential sampling framework to gain valuable insights into the underlying cognitive and neural processes of memory-based decisions. For example, we have learned that the memory bias arises from a single comparison process that is biased towards more vividly remembered options rather than from a mixture of two separate choice strategies (Kraemer et al., 2020). Similarly, by modeling the dynamics of memory-based decisions with an attention-based sequential sampling model (Thomas et al., 2019), we provided evidence that the influence of attention is enhanced when decisions require memory retrieval (Weilbächer et al., 2021). We argue that adopting this framework will remain critical for future research that seeks to close the gap between memory and decision making even further.

One reason for our view is that the framework is compatible with dominant psychological theories of memory-based decision making. Although some scholars have proposed relatively simple decision rules which depend on recognition (Gigerenzer and Gaissmaier, 2011), the information processing paradigm is gaining more and more traction in psychology and neuroscience (Oppenheimer and Kelso, 2015). This paradigm suggests that decision-making draws on fundamental processes of memory, attention, and perception. Decision-relevant information is retrieved (sampled) from an internal representation of the choice options and used to generate a decision outcome. Notably, prominent approaches like Query theory (Johnson et al., 2007) and Decision by Sampling (Stewart et al., 2006) implement this sampling idea, where informative samples drive the decision process towards an eventual decision. Hence, the link to the sequential sampling framework is obvious, and adopting it will be critical to advance the field as it offers a robust mathematical foundation.

Importantly, sequential sampling models are theoretically closely related to action selection processes (Cisek, 2012; Shadlen and Kiani, 2013) and have been related to neural processes in related brain regions such as the frontal eye fields, the pre-supplementary motor area and the posterior parietal cortex in monkeys (Cisek and Kalaska, 2010; Gold and Shadlen, 2007) and human homologues (Gluth et al., 2012, 2013; Hare et al., 2011; Pisauro et al., 2017). Nevertheless, how does value-based evidence accumulation depend on memory processes? While brain imaging studies identified vmPFC and hippocampus as key regions in memory-based decisions (Bakkour et al., 2019; Gluth et al., 2015; Shadlen and Shohamy, 2016), a mechanistic understanding of how information is integrated from memory requires a more nuanced investigation, including real-time observations of how decision processes unfold. Future research may combine sequential sampling models with neuroscientific methods that offer a high temporal resolution such as electro- and magnetoencephalography (EEG/MEG) to identify the relative components of memory retrieval and action selection, allowing a thorough investigation of the underlying cognitive and neural processes.

### 4.3 Memory representation

Up until now, most research has been focused on how decision making is affected by memory processes. Thereby, the clear focus of this research line has been on decision processes, studying how deliberation or valuation works. To understand, however, how memory and decision making interact, we need a better understanding of the involved memory processes. This applies not only to encoding and retrieval processes but also to the representation of information in memory, or *memory structure* (Kahana, 2020). With regard to this question of memory representation, we see two trends gaining more attention in the field of decision making. First, from a connectionist perspective, decision making can be modeled as activation in a neural network with a decision scenario as input and a response as output (Bhatia, 2013; Hunt and Hayden, 2017; Suri et al., 2020). In such a network, memory can be conceptualized as hidden units that have a relatively stable effect on action generating units. While this perspective delivers a memory structure, it does not model a retrieval process with retrieval dynamics. This account holds that value does not need to be represented explicitly but emerges from the activation of the network structure (Yoo and Hayden, 2018).

Another account of memory-based decisions comes from research that uses semantic memory representations such as semantic networks (Siew et al., 2019) or high-dimensional vector spaces (Bhatia et al., 2019). In these accounts, retrieval processes draw on these memory structures and use their information to generate actions. Zhao et al. (2021) and Kraemer et al. (2021) followed this line, demonstrating their applicability to different decision contexts. However, more research in this direction is needed to understand the implications for valuation processes.

### 4.4 Constructing value from memory

While episodic memory often focuses on the retrospective nature of memory, prospective aspects and their potential role in decision-making gained more traction recently (Biderman et al., 2020). It is important to note that the hippocampus and adjacent medial temporal lobe structures do not only enable us to retrieve past episodes, but they also allow us to use such episodes (or parts of them) to construct and envision future states of the world flexibly. In particular, the hippocampus has been shown to be involved in associative value learning (Gerraty et al., 2014; Wimmer and Shohamy, 2012), constructing novel representations of value (Barron et al., 2013) and deliberation during value-based decisions (Bakkour et al., 2019; Bornstein and Daw, 2013).

Importantly, value-based decision making itself has been described as a constructive process (e.g., Lichtenstein and Slovic, 2006). Choice behavior of humans (and other animals) appears to be highly dependent on the context in general and on the available choice set in particular (Busemeyer et al., 2019; Spektor et al., 2021). These findings contradict the idea that people have stable preferences which they need to retrieve in a given choice context. Thus, the neuroeconomic view of decision making as a two-stage process of valuation and action selection is challenged. Instead models that assume preferences to be constructed on the fly so that another, currently available alternative can influence the estimation of an option's value, appear to provide a more complete account of the cognitive process of decision making. At first glance, one could think that the rejection of a simple value-retrieval mechanism may speak against an essential role of memory in decision making. In our view, however, the above-mentioned ability of the brain's memory system to recombine past events for creating future prospects of the world suggests that memory-based processes are also involved in the context-depended formation of preferences. Thus, once more, memory and decision-making processes seem to be intertwined and should be investigated jointly in the future.

## CRediT authorship contribution statement

**Peter M. Kraemer:** Conceptualization, Writing – original draft, Writing – review & editing, Visualization, Project administration. **Regina A. Weilbächer:** Conceptualization, Writing – original draft, Writing – review & editing. **Tehilla Mechera-Ostrovsky:** Conceptualization, Writing – original draft, Writing – review & editing. **Sebastian Gluth:** Conceptualization, Writing – original draft, Writing – review & editing, Supervision, Funding acquisition.

## Declaration of competing interest

The authors declare that they have no known competing financial interests or personal relationships that could have appeared to influence the work reported in this paper.
