# Supplementary material for: Cognitive and neural principles of a memory bias on preferential choices
Source: Curr Res Neurobiol. 2022 Feb 8;3:100029. doi: 10.1016/j.crneur.2022.100029 (PMC9846459; doi:10.1016/j.crneur.2022.100029)
Supplement: Multimedia component 1 [file mmc1.pdf]

Peer Review Overview

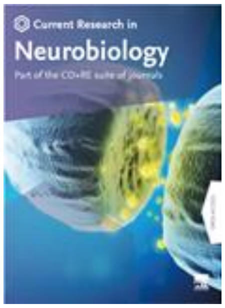

Manuscript Title: Cognitive and Neural Principles of a Memory Bias on Preferential Choices

Received	Aug 25, 2021
1st Decision	Oct 22, 2021
1st Revision Submitted	Dec 22, 2021
2nd Decision	Jan 24, 2022
2nd Revision Submitted	Jan 31, 2022
Accepted	Jan 31, 2022

1st Decision letter

Reference: CRNEUR-D-21-00048
Title: Cognitive and Neural Principles of a Memory Bias on Preferential Choices
Journal: Current Research in Neurobiology

Dear Prof. Dr. Gluth,

Thank you for submitting your manuscript to Current Research in Neurobiology.

I have completed my evaluation of your manuscript. The reviewers recommend reconsideration of your manuscript following major revision. I invite you to resubmit your manuscript after addressing the comments below. Please resubmit your revised manuscript by Dec 11, 2021.

When revising your manuscript, please consider all issues mentioned in the reviewers' comments carefully: please outline in a cover letter every change made in response to their comments and provide suitable rebuttals for any comments not addressed. Please note that your revised submission may need to be re-reviewed.

Current Research in Neurobiology values your contribution and I look forward to receiving your revised manuscript.

CRNEUR aims to be a unique, community-led journal, as highlighted in the Editorial Introduction. As part of this vision, we will be regularly seeking input from the scientific community. We encourage you and your co-authors to take the survey as part of the editorial.

Kind regards,

Anna S Mitchell, Ph.D.
Editor in Chief
Current Research in Neurobiology

Comments from Editors and Reviewers:

Reviewer #1:

The authors present extremely interesting review of the neural and cognitive basis of decision making. The authors discuss the decision making and the role of episodic, semantic and procedural memory processes in decision making, and the adaptation of the process depending on the available information and memory failure. The authors present data from their studies and others suggesting interactions between the hippocampus and vmPFC facilitate decision making, by enabling the use of information concerning the better remembered options,, they also introduce potential roles for the amygdala, orbitofrontal cortex and rostrolateral cortex.

As I said the review is interesting and likely to appeal to a broad readership in cognitive neuroscience. I would suggest, however that the authors have emphasised the cognitive basis of decision making, rather than the neural and so the review is a little one-sided. There is relatively little depth in discussion of the role of different brain regions in either memory or decision making and if this aspect were strengthened then the review would appeal to a wider audience. The authors could consider that the hippocampus is not the only brain region involved in episodic memory, as damage in the vmPFc in humans can also impact this form of memory. It would also be interesting if the authors could increase description and discussion of the role of the amygdala and orbitofrontal cortex on the modulation of HPC-vPFC interaction in memory bias. On a similar vein, in Fig 3, the authors introduce the rostrolateral prefrontal cortex as a region involved in beliefs and value of memory, but provide little supporting data from either their work or the literature.

Reviewer #2:

Kraemer and colleagues present a review of the relationships between value-based decision making and memory, with the purpose of elucidating the interaction between valuation and different forms of memory and their neural substrates. They particularly focus on the contributions of episodic memory to decision making Overall this is an important area of research in cognitive neuroscience, that as the authors rightly point out is often implicitly assumed by researchers of decision making or sometimes only vaguely referred to with little in-depth exploration of its mechanisms. The authors are an authority on the memory/decision making intersections, having conducted a lot of important, systematic, empirical work in the area and having published previous reviews on the topic. The writing was for the most part clear and the ideas easy to follow (although sections 2 and 4 are a little telegraphic, containing many important ideas that are mentioned only in passing and that are not fully supported or explained in the text).
Below we outline some specific suggestions and recommendations, but our main reservation about the review is what seems to us like a great disparity between the stated scope of the manuscript (neural and cognitive mechanisms of memory-based decisions) and what is actually covered in any amount of detail or depth. More than half of the review is devoted to describing a single aspect of the interaction between episodic memory and decision making (the phenomenon of memory bias), using a single task to measure its possible underlying neurocognitive mechanisms (remember and decide task). The authors state the review focuses on episodic memory, but despite this disclaimer, often appear to draw conclusions or discuss the role of memory in decision making more broadly. Although no doubt the memory bias is an important phenomenon, and the authors ought to be congratulated on their systematic research program of it, it only captures some aspects of episodic memory/decision making interactions. Moreover, as the authors rightly point out, cognitive processes can only be inferred from

behavior; the focus on a single lab-based paradigm to study memory bias allows for systematic manipulations of variables, but also significantly limits the generalizability of findings. Therefore, we think that if the authors wish to maintain the current focus of the review, other sections including the title, abstract introduction and conclusions need to be revised to reflect the much more limited scope: episodic memory bias as captured by the remember and decide task. Alternatively, the authors could consider widening the review scope to other aspects of mnemonic influences on decision making which would probably need to come at the expense of some of the detail describing the results from the remember and decide task.

Comments/recommendations:
- A host of prior experiences are noted as influencing decision making (procedural memory, semantic memory, episodic memory) and there are likely others (e.g. conditioned responses, high-order conditioned response, implicit biases). It may be useful to explain why focus specifically on episodic memory and its failure during decision making. Most of human behavior (and decision making) is not reliant on episodic memory but on other prior experiences (habits, semantics, schemas). Moreover, because of the nature of the remember and decide task, episodic memory failures can only be compensated by other (available) episodic memories. Contrary to real life, within this task participants are facing decisions with a clean sheet (arbitrary value-location associations are only learned in the lab). In real life, in cases where a decision needs to be made based on an episodic memory and one cannot be recalled, humans are more likely to resort to decisions based on habits, schemas or semantics, or their decisions might be biased by implicit conditioned responses, rather than choosing another episodically remembered unattractive choice. This speaks to the generalization limitation of relying on a single lab-based task to make assertions about mnemonic processes in decision making.
- Within the proposed framework, memory is treated as a meta-cognitive process applied to decision making (choice) and some research into meta-cognitive beliefs about memory is described. The latter has been extensively studied in the memory domain and a term that is highly relevant here is meta-mnemonic processes ('memory decision making'). These serve to make decisions about the amount of mnemonic evidence available, setting decision criteria of whether or not to use the evidence for driving behavior etc. These tend to rely on the same neurocognitive mechanisms described here for making value decision (e.g. vmPFC-HPC axis). Some reference to that literature seems pertinent, and perhaps clarification of whether the neural and behavioral patterns observed necessarily relate to item value decisions or to memory availability decisions (or both). For example, U shaped relationships described in section 3.3 have been described before in both the valuation and memory domains in both imaging (Lebreton et al. 2015) and lesion (Hebscher et al., 2016) research and appear to reflect a meta-cognitive control function (e.g. confidence) mediated by vmPFC. The U shaped relationship described in the paper as reflected memory per se may in fact reflect (meta mnemonic) confidence and decision making processes applied to memory.
- (Section 2.3) Suggesting that the role of medial OFC in value representation was discovered by human neuroimaging less than 2 decades ago is misleading. It has been known since the 1980's at least through lesion and electrophysiology in animals (e.g. Rolls) and human lesion studies have also highlighted its role in value representation and decision making.
- (Section 2.4) Procedural memory is only one form of non-declarative memory. Others include priming, conditioning and non-associative learning such as habituation. The inclusion oof the Wimmer and Shohamy HPC finding as reflecting its involvement in procedural memory is inaccurate. Their study was investigating sensory pre-conditioning.
- The suggestion that the mechanism for memory bias is a heuristic choice process needs further clarification. Currently it sounds like the word bias was replaced by the word heuristic.

Specific details that may help clarify the manuscript:

Section 2
- For general readership some key terms being defined, or their significance described (e.g., expected value, expected utility). What is the difference between expected value vs. expected utility? How is utility a function of an individual's wealth? Why is this important? If it isn't, consider removing. Similarly, for behaviorism.
- Define signal detection theory beyond just saying that it allows for separation between perceptual and decision-making process on a cognitive level. How does this relate to perceptual judgments? How are perceptual judgements linked to decision making?
- Define Action Selection and how it is an integral part of decision making, beyond neural underpinning. The assertion that neurons represent different action plans with their neural activity needs supporting citations and the circuitry of value-based action selection needs to be better described.
- Define how value fits into a wider decision making framework define different types of value (e.g., subjective, objective) since these play an important role in the studies that follow.
- When introducing vmPFC, mOFC, and ventral striatum the role of the ventral striatum also needs to be defined to explain why it was referenced.
- Define goods space and action space more clearly and how they are related.
- The term agent should be defined for readers unfamiliar with decision making frameworks. This could be resolved via early suggestion to include a well defined decision making framework when introducing action selection and value. Similarly choice set generation should be better defined and elaborated on.
- Episodic memory and Autobiographical memory are used interchangeably. They are not, both as cognitive constructs and at the neural level.
Section 3
- Diagram of task design could also include estimate-your-memory task since this is referenced below.

- Define primacy & recency effects, associative priming, acceptance and avoidance of risk and risk aversion. A lot is being asked of the reader to understand the relevance here.
- Memory strength is discussed throughout the paper, but not how it was tested, unless the authors mean memory failures, which is different (see above comment on criterion setting etc.).
- What are psychological institutions? How do these differ from cognitive mechanisms?
Section 4
- Change in language back to valuation and action selection feels abrupt. Consider using this language while describing studies above.
- The interpretation of striatal activation during successful high confidence memory retrieval introduces a new theory that has not been discussed before (and is related to the above comment on memory decision making). This should probably be expanded on (or omitted?).
- Consider renaming Mathematical modeling of choices from memory since it is predominantly interested in promoting sequential sampling framework and not mathematical modelling in general.

1st Author Response Letter

Response to comments from Editors and Reviewers:

Comments from Reviewer 1

The authors present extremely interesting review of the neural and cognitive basis of decision making. The authors discuss the decision making and the role of episodic, semantic and procedural memory

processes in decision making, and the adaptation of the process depending on the available information and memory failure. The authors present data from their studies and others suggesting interactions between the hippocampus and vmPFC facilitate decision making, by enabling the use of information concerning the better remembered options,, they also introduce potential roles for the amygdala, orbitofrontal cortex and rostrolateral cortex.

We thank the reviewer for this very positive evaluation of our work.

As I said the review is interesting and likely to appeal to a broad readership in cognitive neuroscience. I would suggest, however that the authors have emphasised the cognitive basis of decision making, rather than the neural and so the review is a little one-sided. There is relatively little depth in discussion of the role of different brain regions in either memory or decision making and if this aspect were strengthened then the review would appeal to a wider audience. The authors could consider that the hippocampus is not the only brain region involved in episodic memory, as damage in the vmPFc in humans can also impact this form of memory. It would also be interesting if the authors could increase description and discussion of the role of the amygdala and orbitofrontal cortex on the modulation of HPC-vPFC interaction in memory bias. On a similar vein, in Fig 3, the authors introduce the rostrolateral prefrontal cortex as a region involved in beliefs and value of memory, but provide little supporting data from either their work or the literature.

We agree with the reviewer that we put a stronger focus on the cognitive rather than the neural basis of memory-based decisions. Note that in response to another reviewer, we clarified the scope of this review by emphasizing our work on the memory bias. The majority of our research on the memory bias dealt with its cognitive mechanisms (including detailed cognitive modeling), which necessarily puts a stronger focus on the cognitive compared to the neural side.

Having said this, we followed the reviewer's suggestions to elaborate on findings relating to the role of different brain regions in memory and decision making. In the last paragraph of Section 2.2, we discuss the potential role of the parietal lobe / posterior parietal cortex in memorybased decisions as another region linked to episodic memory, and we emphasize that the vmPFC contributes not only to value-based decisions but episodic memory, too. Furthermore, in section 4.1, we extend our discussion about the potential roles of the rostrolateral prefrontal cortex in mediating meta-cognitive influences on the memory bias as well as the amygdala and OFC in mediating uncertainty-related influences. In this context, we suggest using Dynamic Causal Modeling (DCM) (Friston et al., 2003) in future neuroimaging studies to test for modulations of the hippocampus-vmPFC connectivity by third regions such as amygdala or OFC.

Comments from Reviewer 2

Kraemer and colleagues present a review of the relationships between valuebased decision making and memory, with the purpose of elucidating the interaction between valuation and different forms of memory and their neural substrates. They particularly focus on the contributions of episodic memory to decision making Overall this is an important area of research in cognitive neuroscience, that as the authors rightly point out is often implicitly assumed by researchers of decision making or sometimes only vaguely referred to with little in-depth exploration of its mechanisms. The authors are an authority on the memory/decision making intersections, having conducted a lot of important, systematic, empirical work in the area and having published previous reviews on the topic. The

writing was for the most part clear and the ideas easy to follow (although sections 2 and 4 are a little telegraphic, containing many important ideas that are mentioned only in passing and that are not fully supported or explained in the text). Below we outline some specific suggestions and recommendations, but our main reservation about the review is what seems to us like a great disparity between the stated scope of the manuscript (neural and cognitive mechanisms of memory-based decisions) and what is actually covered in any amount of detail or depth. More than half of the review is devoted to describing a single aspect of the interaction between episodic memory and decision making (the phenomenon of memory bias), using a single task to measure its possible underlying neurocognitive mechanisms (remember and decide task). The authors state the review focuses on episodic memory, but despite this disclaimer, often appear to draw conclusions or discuss the role of memory in decision making more broadly. Although no doubt the memory bias is an important phenomenon, and the authors ought to be congratulated on their systematic research program of it, it only captures some aspects of episodic memory/decision making interactions. Moreover, as the authors rightly point out, cognitive processes can only be inferred from behavior; the focus on a single lab-based paradigm to study memory bias allows for systematic manipulations of variables, but also significantly limits the generalizability of findings. Therefore, we think that if the authors wish to maintain the current focus of the review, other sections including the title, abstract introduction and conclusions need to be revised to reflect the much more limited scope: episodic memory bias as captured by the remember and decide task. Alternatively, the authors could consider widening the review scope to other aspects of mnemonic influences on decision making which would probably need to come at the expense of some of the detail describing the results from the remember and decide task.

We thank the reviewers for their detailed evaluation of our work. Their comments helped us to address several shortcomings of our previous manuscript, allowing us to submit a significantly improved revision.

We agree that there was a disparity with respect to the scope of the review. The central purpose of our review is (and has been) to embed our research on the memory bias in the field of memory-based decision making. Based on the reviewers' feedback, we realized that it is important to be more explicit about our central aims. Therefore, we adjusted large parts of the manuscript including the title and the abstract and Section 2, which we revised substantially, to make the focus of this review more evident.

A host of prior experiences are noted as influencing decision making (procedural memory, semantic memory, episodic memory) and there are likely others (e.g. conditioned responses, high-order conditioned response, implicit biases). It may be useful to explain why focus specifically on episodic memory and its failure during decision making. Most of human behavior (and decision making) is not reliant on episodic memory but on other prior experiences (habits, semantics, schemas). Moreover, because of the nature of the remember and decide task, episodic memory failures can only be compensated by other (available) episodic memories. Contrary to real life, within this task participants are facing decisions with a clean sheet (arbitrary value-location associations are only learned in the lab). In real life, in cases where a decision needs to be made based on an episodic memory and one cannot be recalled, humans are more likely to resort to decisions based on habits, schemas or semantics, or their decisions might be biased by implicit conditioned responses, rather than choosing another episodically remembered unattractive choice. This speaks to the generalization limitation of relying on a single lab-based task to make assertions about mnemonic processes in

decision making.

We agree with the reviewers that other memory systems play important roles in everyday decision making. It was not our purpose to suggest that episodic memory would be more important than, say, procedural or semantic memory. In the revised manuscript (Section 2.2, 3rd paragraph), we take model-free reinforcement learning as an example and acknowledge that a lot of effort has been put into studying the role this form of learning on decision making. At the same time, however, we note that the role of episodic memory in deliberative decision making has been emphasized in recent years, leading even to the proposal of ”episodic reinforcement learning” (Bornstein et al., 2017), according to which people may retrieve specific episodes of rewarding or punishing events when making decisions in learning contexts. We hope that our updated version makes clear why episodic is of special interest in neuroeconomic decision-making, while agreeing at the same time, that other memory systems certainly play important roles, too.

Within the proposed framework, memory is treated as a meta-cognitive process applied to decision making (choice) and some research into meta-cognitive beliefs about memory is described. The latter has been extensively studied in the memory domain and a term that is highly relevant here is meta-mnemonic processes (’memory decision making’). These serve to make decisions about the amount of mnemonic evidence available, setting decision criteria of whether or not to use the evidence for driving behavior etc. These tend to rely on the same neurocognitive mechanisms described here for making value decision (e.g. vmPFC-HPC axis). Some reference to that literature seems pertinent, and perhaps clarification of whether the neural and behavioral patterns observed necessarily relate to item value decisions or to memory availability decisions (or both). For example, U shaped relationships described in section 3.3 have been described before in both the valuation and memory domains in both imaging (Lebreton et al. 2015) and lesion (Hebscher et al., 2016) research and appear to reflect a meta-cognitive control function (e.g. confidence) mediated by vmPFC. The U shaped relationship described in the paper as reflected memory per se may in fact reflect (meta mnemonic) confidence and decision making processes applied to memory.

The reviewers point to an intriguing explanation of the findings reported in our work on how meta-cognitive beliefs drive the memory bias on choice. Both, confidence and meta-memory performance exhibit a U-shaped relationship with value and accuracy, respectively. This is also reflected by the fact that vmPFC activity appears to scale with both (linear) value and (quadratic) confidence (Lebreton et al., 2015). Lesions to vmPFC (and posterior OFC) seem to disrupt the quadratic relationship between meta-mnemonic judgements and actual performance (Hebscher et al., 2016). Taken together, these results seem to indicate that the memory bias is tightly linked to the notion of confidence on both the cognitive and the neural level. More specifically, when choosing between a remembered and a forgotten option, people may take into account whether they have sufficient memory strength for choosing an option. Because this memory strength (or confidence) appears to be lowest for options that are slightly below average in terms of subjective value (Mechera-Ostrovsky & Gluth, 2018), the decision is most difficult at this point, which is in line with people being indifferent between the two options. We elaborate on these ideas in the revised manuscript (last paragraph of Section 3.3), discussing and citing the highly relevant work mentioned by the reviewers.

(Section 2.3) Suggesting that the role of medial OFC in value representation was discovered by human neuroimaging less than 2 decades ago is misleading. It has been known since the 1980’s at least

through lesion and electrophysiology in animals (e.g. Rolls) and human lesion studies have also highlighted its role in value representation and decision making.

Thanks for pointing this out. In the revised manuscript (now in the 2nd paragraph of Section 2.1), we mention the fact that relevant electrophysiological and lesion studies were already performed in the 1980’s and cite (more recent) reviews of these studies, including a review by Edmund Rolls.

(Section 2.4) Procedural memory is only one form of non-declarative memory. Others include priming, conditioning and non-associative learning such as habituation. The inclusion of the Wimmer and Shohamy HPC finding as reflecting its involvement in procedural memory is inaccurate. Their study was investigating sensory pre-conditioning.

In principle, we agree with the reviewers that more fine-grained specifications of non-declarative memory are possible. However, when adapting the manuscript to put a clearer focus on the memory bias (see above), we dropped the discussion points that are relevant to this comment. Nonetheless, we make clear (in Section 2.2, paragraph 2) that non-declarative memory includes the memory systems mentioned, based on the standard taxonomy which is depicted in Milner et al., 1998.

The suggestion that the mechanism for memory bias is a heuristic choice process needs further clarification. Currently it sounds like the word bias was replaced by the word heuristic.

We apologize for the lack of clarity regarding the dissociation of bias and heuristic. The memory bias is the empirical phenomenon that people prefer remembered over forgotten options. The heuristic choice process we discuss is (a part of) one potential computational account which may explain the memory bias (however, our analyses and modeling of response times speak against this account). We adapted the text (1st paragraph of Section 3.6) and hope that the distinction of bias and heuristic is clearer now.

Specific details that may help clarify the manuscript:

Section 2

For general readership some key terms being defined, or their significance described (e.g., expected value, expected utility). What is the difference between expected value vs. expected utility? How is utility a function of an individual’s wealth? Why is this important? If it isn’t, consider removing. Similarly, for behaviorism.

We thank the reviewers for pointing out that these terms were not defined appropriately. When narrowing the scope of the review, we decided to focus on the main message of this section that is concerned with the notion subjective value. In the first paragraph of Section 2.1, we briefly mention the term utility and make clear that is equivalent to the term subjective value, which specify in more detail and explain why it is relevant for neuroeconomics in general and for the current work in particular. The topics of expected value, expected utility etc. are, as the reviewers point out, not critical for this.

Define signal detection theory beyond just saying that it allows for separation between perceptual and decision-making process on a cognitive level. How does this relate to perceptual judgments? How are perceptual judgements linked to decision making?

Similar to the previous point, we took out the topic of SDT, as it is not so relevant for our discussion of the memory bias.

Define Action Selection and how it is an integral part of decision making, beyond neural underpinning. The assertion that neurons represent different action plans with their neural activity needs supporting citations and the circuitry of value-based action selection needs to be better described.

In the revised manuscript (3rd paragraph of Section 2.1), we define action selection as the selection among action plans to achieve a particular goal, much in line with a key reference in this domain (Cisek & Kalaska, 2010). The work by Cisek and Kalaska also show that neural populations in the considered regions are tuned to specific actions due to their efferent connections with the motor system. We describe this circuitry in more detail in the relevant section and outline key references for the process of action selection.

Define how value fits into a wider decision making framework define different types of value (e.g., subjective, objective) since these play an important role in the studies that follow.

See above (we restrict the discussion of value around the topic of subjective value, which we explicate in more detail).

When introducing vmPFC, mOFC, and ventral striatum the role of the ventral striatum also needs to be defined to explain why it was referenced.

After restructuring the manuscript, the part in which we introduce both vmPFC and ventral striatum have been taken out. When reporting the fMRI results of our study in paragraph 2 of Section 3.2, however, we mention that subjective value signals were also found in the striatum and briefly explain that activity in both vmPFC and ventral striatum are often correlated with subjective value, which seems plausible given that both regions are major projection areas of the dopaminergic reward system of the brain.

Define goods space and action space more clearly and how they are related.

Since the terms themselves are not so relevant for our research, we omitted them but rewrote the paragraph at the end of Section 2.1 to make the problem clear: It is unclear whether value comparison occurs in regions that code for value irrespective of the actions that are need to obtain a specific option (good space), or whether it only occurs in action-selective regions (action space).

The term agent should be defined for readers unfamiliar with decision making frameworks. This could be resolved via early suggestion to include a well defined decision making framework when introducing action selection and value. Similarly choice set generation should be better defined and

elaborated on.

To avoid confusion, we decided to always use the term ”decision-maker” instead of ”agent”, and we do not mention ”choice set generation” anymore (since we took out the paragraph on semantic memory).

Episodic memory and Autobiographical memory are used interchangeably. They are not, both as cognitive constructs and at the neural level.

Thanks for pointing this out. In the manuscript, we refer to episodic memory since it is believed that mnemonic information from retrieved episodes are weighed in decision processes (Biderman et al., 2020). Thereby, autobiographical episodes may play a role. However, as the reviewers rightfully point out, autobiographical memory is a different cognitive construct since it only applies to factual information (Wheeler et al., 1997) and is associated with different prefrontal activity patterns (Gilboa, 2004). We adapted the manuscript in the second paragraph of Section 2.2 to make clear that we focus on episodic memory rather than autobiographical memory.

Section 3 Diagram of task design could also include estimate-your-memory task since this is referenced below.

We thank the reviewers for this suggestion but decided against adding this task, as it was only used in one of our studies and is not an integral part of the remember-and-decide task.

Define primacy & recency effects, associative priming, acceptance and avoidance of risk and risk aversion. A lot is being asked of the reader to understand the relevance here.

In the relevant section (beginning of Section 3), we make sure that all of these terms are defined (the terms acceptance and avoidance of risk are dropped, but we still use (and define) risk aversion).

Memory strength is discussed throughout the paper, but not how it was tested, unless the authors mean memory failures, which is different (see above comment on criterion setting etc.).

By memory strength, we mean the subjective estimation of vividness of information in memory. We have assessed this variable during our experiments in the Recall phase of the remember-and-decide task (after each recall, participants were asked to judge their memory strength of the to-be-recalled item). In the revised manuscript, we have added it to Figure 1 and discuss it when explaining the task in the 2nd paragraph of Section 3.1.

What are psychological institutions? How do these differ from cognitive mechanisms?

By psychological intuitions we meant (somewhat obvious) folk-psychological reasons for why people exhibit a memory bias. When presenting our work at conferences, we often experienced other researchers proposing quite spontaneously that the memory bias could be related to uncertainty or

beliefs. However, we realize that these are just anecdotes, so we decided to omit this term in the revised manuscript.

Section 4 Change in language back to valuation and action selection feels abrupt. Consider using this language while describing studies above.

Section 3 contains the terms of action selection and valuation where appropriate. Since most studies are rather cognitive and action selection and valuation are neural processes, we agree that these terms do not occur frequently in Section 3. However, it is important to maintain this wording during Section 4, where we discuss the memory bias results presented in Section 3 and their relation to action selection and valuation.

The interpretation of striatal activation during successful high confidence memory retrieval introduces a new theory that has not been discussed before (and is related to the above comment on memory decision making). This should probably be expanded on (or omitted?).

We would not fully agree that the observation of a link between striatal activation and high confidence in memory introduces an entire new theory. Yet, we extended this point to make the link between this finding and our phenomenon of interest (i.e., the memory bias) more transparent (see last paragraph of Section 4.1).

Consider renaming Mathematical modeling of choices from memory since it is predominantly interested in promoting sequential sampling framework and not mathematical modelling in general.

We agree with this notion and adapted the wording accordingly. The title of this sub-section is now ”Sequential sampling modeling of choices from memory”.

*References

Biderman, N., Bakkour, A., & Shohamy, D. (2020). What Are Memories For? The Hippocampus Bridges Past Experience with Future Decisions. Trends in Cognitive Sciences, 24 (7), 542–556. https://doi.org/10.1016/j.tics.2020.04.004

Bornstein, A. M., Khaw, M. W., Shohamy, D., & Daw, N. D. (2017). Reminders of past choices bias decisions for reward in humans. Nature Communications, 8 (May), 1–9. https://doi.org/10.1038/ncomms15958

Cisek, P., & Kalaska, J. F. (2010). Neural mechanisms for interacting with a world full of action choices. Annual Review of Neuroscience, 33, 269–298. https: / / doi. org / 10. 1146/annurev.neuro.051508.135409

Friston, K. J., Harrison, L., & Penny, W. (2003). Dynamic causal modelling. NeuroImage, 19 (4), 1273–1302. https://doi.org/10.1016/S1053-8119(03)00202-7

Gilboa, A. (2004). Autobiographical and episodic memory - One and the same? Evidence from prefrontal activation in neuroimaging studies. Neuropsychologia, 42 (10), 1336– 1349. https://doi.org/10.1016/j.neuropsychologia.2004.02.014

Hebscher, M., Barkan-Abramski, M., Goldsmith, M., Aharon-Peretz, J., & Gilboa, A. (2016). Memory, Decision-Making, and the Ventromedial Prefrontal Cortex (vmPFC): The Roles of Subcallosal and Posterior Orbitofrontal Cortices in Monitoring and Control Processes. Cerebral Cortex, 26, 4590–4601. https://doi.org/10.1093/cercor/bhw220

Lebreton, M., Abitbol, R., Daunizeau, J., & Pessiglione, M. (2015). Automatic integration of confidence in the brain valuation signal. Nature Neuroscience, 18 (8), 1159–1167. https://doi.org/10.1038/nn.4064

Mechera-Ostrovsky, T., & Gluth, S. (2018). Memory beliefs drive the memory bias on valuebased decisions. Scientific reports, 8 (1), 1–10.

Milner, B., Squire, L. R., & Kandel, E. R. (1998). Cognitive neuroscience and the study of memory. Neuron, 20 (3), 445–468. https://doi.org/10.1016/S0896-6273(00)80987-3

Wheeler, M. A., Stuss, D. T., & Tulving, E. (1997). Toward a theory of episodic memory: The frontal lobes and autonoetic consciousness. https: / / doi. org / 10. 1037 / /0033 - 2909.121.3.331

2nd Decision letter

Reference: CRNEUR-D-21-00048
Title: Cognitive and Neural Principles of a Memory Bias on Preferential Choices
Journal: Current Research in Neurobiology

Dear Prof. Dr. Gluth,

Thank you for submitting your manuscript to Current Research in Neurobiology.

I have completed my evaluation of your manuscript. I would like to accept your manuscript, but first, please may I request that you make two further minor revisions (as detailed below) and consider revising some of the jargon within the text. I invite you to resubmit your manuscript after addressing the comments below. Please resubmit your revised manuscript by Feb 08, 2022.

When revising your manuscript, please consider all issues mentioned in the reviewers' comments carefully: please outline in a cover letter every change made in response to their comments and provide suitable rebuttals for any comments not addressed. Please note that your revised submission may need to be re-reviewed.

Current Research in Neurobiology values your contribution and I look forward to receiving your revised manuscript.

CRNEUR aims to be a unique, community-led journal, as highlighted in the Editorial Introduction. As part of this vision, we will be regularly seeking input from the scientific community. We encourage you and your co-authors to take the survey as part of the editorial.

Kind regards,

Anna S Mitchell, Ph.D.
Editor in Chief
Current Research in Neurobiology

Comments from Editors and Reviewers:

Thank you for your revisions and thorough explanations. Your manuscript has improved considerably. Myself and the available reviewer both agree that this review will make a valuable addition to the literature.

Minor revisions:
1) Please can you briefly explain each of the figures in the graphical abstract. You can do this within the graphical abstract with a simple informative title for each image.
2) At the top of page six, there is a mix of studies discussing vmPFC, then parietal cortex, then mPFC, then vmPFC again. Please can this be reworded for better clarity.

Reviewer #2:

I very much appreciate the responsiveness of the authors to the reviews. I think the current version is much improved, and provides a more balanced account of the place of the described work within a more extensive literature. I think removal of a lot of jargon will make it more accessible to a wider readership. This will make a nice addition to the literature.

2nd Author Response Letter

Response to comments from Editors and Reviewers:

Thank you for the opportunity to revise and resubmit our manuscript to Current Research in Neurobiology. We are glad that you and the reviewers were very content with the first revision. In the new revision, we have addressed your remaining two points. Specifically, we rearranged and rephrased the paragraph on the role of vmPFC and other regions in memory processes and memory-based decisions. Now, we do not switch back and forth anymore in discussing those different brain regions. Also, we added informative titles to the graphical abstract as suggested.

Accept Letter

Dear Prof. Dr. Gluth,

Thank you for submitting your manuscript to Current Research in Neurobiology.

I am pleased to inform you that your manuscript has been accepted for publication. Congratulations to you.

Your accepted manuscript will now be transferred to our production department. We will create a proof which you will be asked to check, and you will also be asked to complete a number of online forms required for publication. If we need additional information from you during the production process, we will contact you directly.

We appreciate and value your contribution to Current Research in Neurobiology. We regularly invite authors of recently published manuscript to participate in the peer review process. If you were not

already part of the journal's reviewer pool, you have now been added to it. We look forward to your continued participation in our journal, and we hope you will consider us again for future submissions.

CRNEUR aims to be a unique, community-led journal, as highlighted in the Editorial Introduction. As part of this vision, we will be regularly seeking input from the scientific community and encourage you and your co-authors to take the survey.

Kind regards,

Anna S Mitchell, Ph.D.
Editor in Chief
Current Research in Neurobiology

-------- End of Review Comments --------
